# The Variable Origin of the Recurrent Artery of Heubner: An Anatomical and Morphometric Study

**DOI:** 10.1155/2013/873434

**Published:** 2013-07-09

**Authors:** Hisham El Falougy, Petra Selmeciova, Eliska Kubikova, Zora Haviarová

**Affiliations:** Institute of Anatomy, Faculty of Medicine, Comenius University, Spitalska 24, 813 72 Bratislava, Slovakia

## Abstract

The recurrent artery of Heubner (RAH) is the largest vessel of the medial lenticulostriate arteries. It supplies many deep structures, mainly the corpus striatum, the globus pallidus, and the anterior crus of the internal capsule. The aim of the present paper was studying the morphological variations of the RAH and its diameter in relation to different areas of origin. The series contained the records from 183 formalin-fixed adult human brains. The calibrated digital images of the studied brains were evaluated and measured by Image J, which can calculate the number of pixels and convert them to metric measures. The RAH arose most often from the postcommunicating part of the anterior cerebral artery (47.81%). It originated from the precommunicating part of the anterior cerebral artery in 3.55% and at the level of the anterior communicating artery in 43.4% of cases. The RAH was missing in 5.19% and doubled in 6.28% of cases. The mean outer diameter of the RAH was 0.6 mm. The maximal measured diameter was 1.34 mm, and the minimal diameter was 0.19 mm. The awareness of the various anatomical and morphometric variations of the RAH is essential in planning the neurosurgical procedures to avoid unexpected neurological complications.

## 1. Introduction

Since 1872, when the German pediatrician Johann Otto Leonhard Heubner described a constant small artery arising from the base of the anterior cerebral artery and supplying blood to the head of the corpus striatum [1, 2], that vessel has gained the attention of many researchers. Various terms were used to sign this artery. It was named as the anterior striate artery, the long telencephalic artery, or the long centralis artery [3]. The latest anatomical nomenclature marked the vessel as the distal medial striate artery [4]. Aitken (1909) labeled the vessel for the first time as Heubner's artery. Joseph Shelleshear elaborated the well-known term “recurrent artery of Heubner” in 1920 [1].

The surgical terminology is dividing the anterior cerebral artery (ACA) into A1—precommunicating portion; A2—from the anterior communicating artery (ACoA) until the callosomarginal artery; and A3—distal to callosomarginal artery [5]. The central or the perforating arteries are small branches of the circle of Willis. These arteries are supplying the deep structures of the brain. They penetrate the brain mostly at the anterior or the posterior perforating substances [5].

The recurrent artery of Heubner (RAH) is usually the largest of the perforating medial lenticulostriate arteries branching from ACA. The RAH is branching from A1, from A2, or at the junction of ACA-ACoA [2, 6]. Later the artery turns posteriorly and runs parallel and is anterior to A1. It penetrates the lateral portion of the anterior perforating substance [7]. The course of the RAH is closely related to the posterior portion of the orbitofrontal cortex and mainly to the gyrus rectus [6, 8]. The artery passes inferiorly and laterally to the origin of olfactory striae before reaching the anterior perforating substance [3].

The RAH supplies blood to the medial portion of the orbitofrontal cortex, the anterior portion of the caudate nucleus, the anterior third of the putamen, the external segment of the globus pallidus, and the anterior crus of the internal capsule [1, 2, 6, 8, 9]. The artery also supplies the olfactory region, the anterior hypothalamus, the nucleus accumbens, parts of the uncinate fasciculus, the diagonal band of Broca, and the basal nucleus of Meynert [3, 8, 10–12].

The anatomical variation of RAH is related to its number, presence, or absence, and the diverse origin from ACA is of considerable clinical impact mainly from the point of view of the surgical procedures involving the anterior portion of the circle of Willis or the topographically related structures. The aim of this work was to study the anatomical anomalies of the RAH and its diameter in relation to different points of origin.

## 2. Methods

The work contains records from 183 adult human brains (366 hemispheres) obtained from the Institute of Anatomy, Faculty of Medicine, Comenius University in Bratislava. The specimens were dissected in the period from 2002 to 2010. Following their removal from the cranial cavity, they were fixed in solution of formalin and benzyl alcohol. The arachnoid was carefully removed from the base of each brain to assess the circle of Willis and its branches. The dissected brains were documented with an Olympus digital camera (model: Camedia C-5050). The images were calibrated by applying a plastic ruler in situ. We used the image processing software Image J (U.S. National Institutes of Health) to study and analyze the images. The calibrated ruler formed a spatial scale to determine the known metric measure in each image. According to these data Image J was able to determine the image distances in metric unit by calculating the pixel differences.

The RAH point of origin, possible abnormalities or variations, and its external diameter were evaluated and analyzed in each brain. Due to the fact that the measurements were carried out on formalin-fixed brains, we have to calculate with 5–10% reduction in vascular diameter [2].

## 3. Results

**Figure 1:**
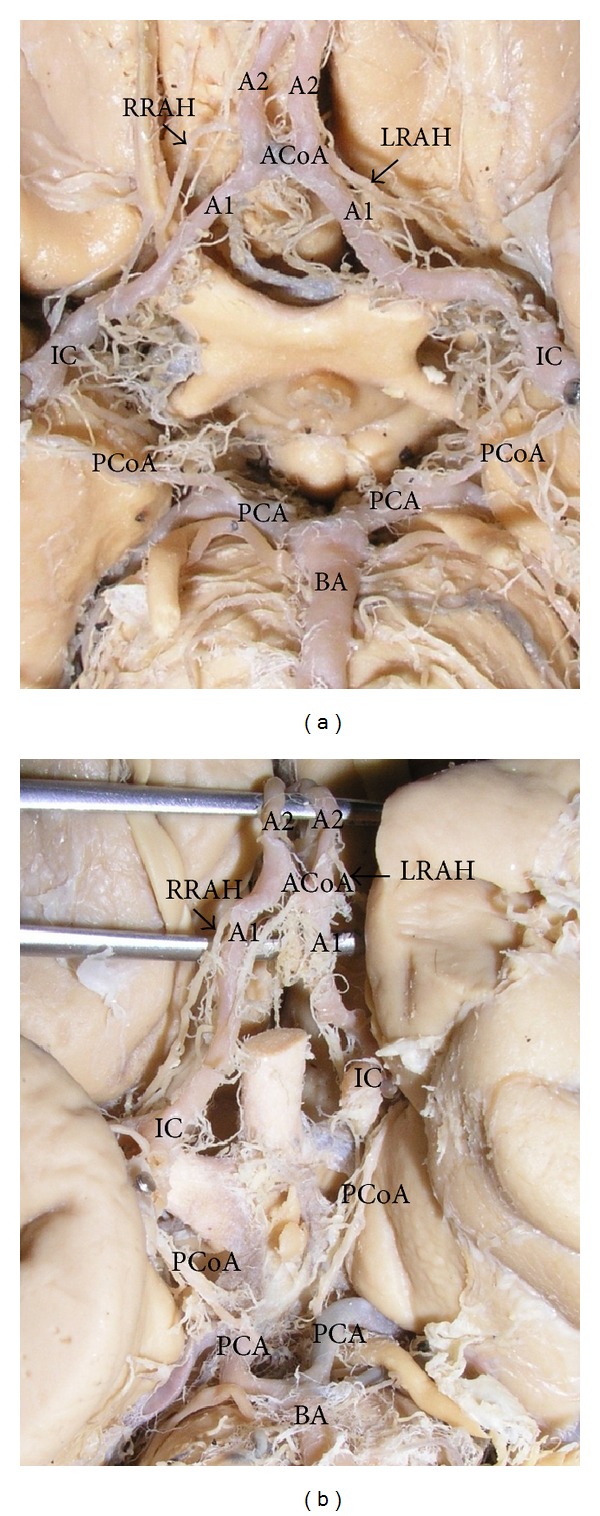
The areas of origin of the recurrent artery of Heubner. (a) The right recurrent artery of Heubner (RRAH) arose from A2; the left vessel (LRAH) originated at the junction of ACA-ACoA. (b) The right recurrent artery of Heubner (RRAH) originated from A1; the left vessel (LRAH) arose from A2. A1—precommunicating part of anterior cerebral artery (ACA); A2—postcommunicating part of ACA; ACoA—anterior communicating artery; IC—internal carotid artery; PCoA—posterior communicating artery; PCA—posterior cerebral artery; BA—basilar artery.

The RAH originated from the precommunicating portion—A1—in 13 hemispheres (3.55% of cases). The A2 was the portion of origin of the artery in 175 hemispheres (47.81% of cases). The artery branched at the junction of ACA-ACoA in 159 hemispheres (43.4% of cases). The RAH was missing in 19 hemispheres (5.19% of cases) (Figures 1, 3(a), 3(b), and 3(c)).

**Figure 2:**
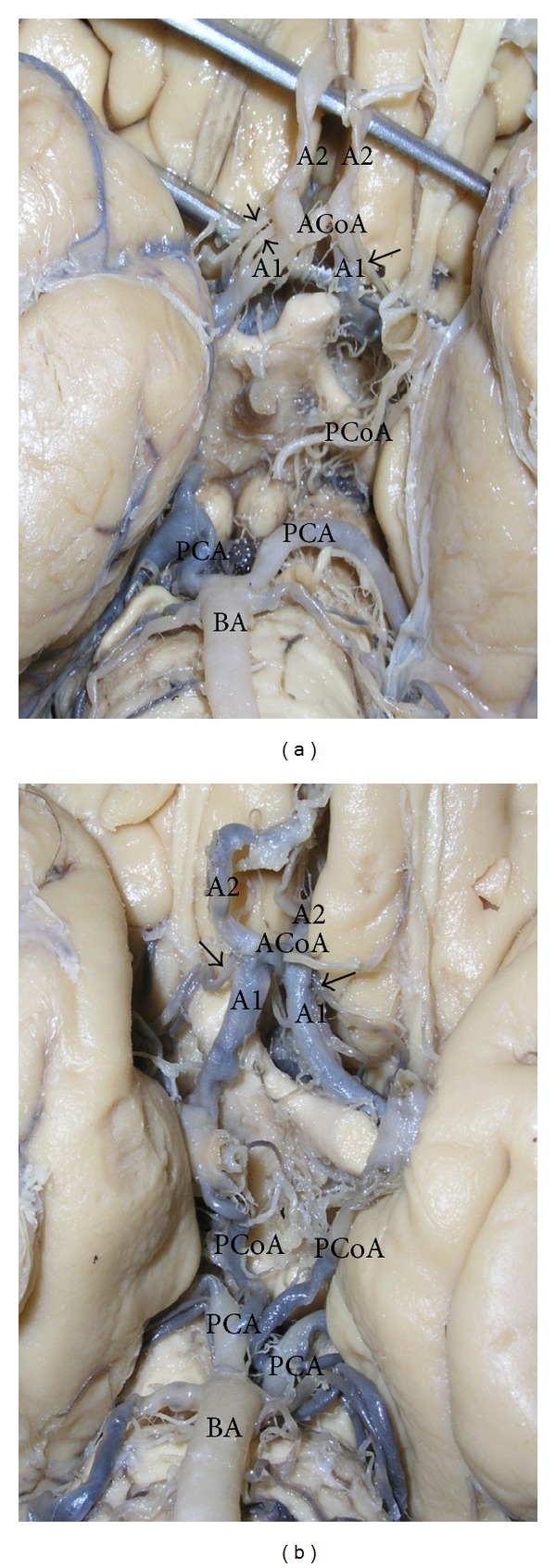
Double recurrent artery of Heubner (RAH). (a) Double RAH with different points of origin (arrow heads); single RAH (arrow). (b) Bilateral doubled RAH originating as one stem and later bifurcated (arrow). A1—precommunicating part of anterior cerebral artery (ACA); A2—postcommunicating part of ACA; ACoA—anterior communicating artery; IC—internal carotid artery; PCoA—posterior communicating artery; PCA—posterior cerebral artery; BA—basilar artery.

**Figure 3:**
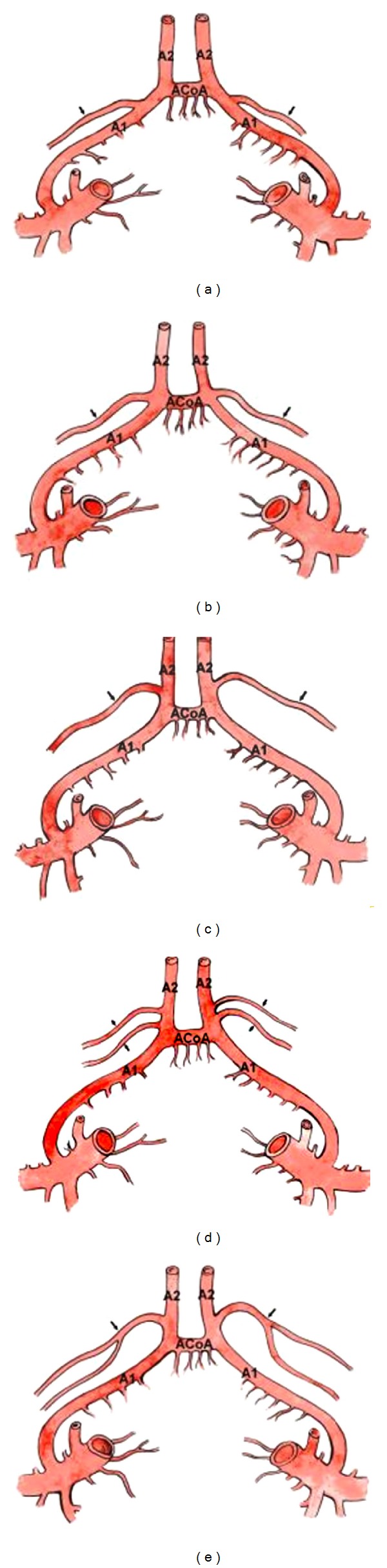
(a), (b), and (c) illustrate the areas of origin of the recurrent artery of Heubner (black arrow). (a) The RAH originated from the precommunicating part (A1) of the anterior cerebral artery. (b) The RAH originated at the level of the anterior communicating artery (ACoA). (c) The RAH originated from the postcommunicating part (A2) of the anterior cerebral artery. (d) and (e) illustrate the double recurrent artery of Heubner. (d) The double RAH originated from different points. (e) The double RAH originated as one stem, which later bifurcated.

The RAH was single in 88.5% of cases (324 hemispheres) and doubled in 6.28% of cases (23 hemispheres). The artery was bilaterally doubled in 5 brains. We observed unilateral duplication of the vessel in 13 hemispheres. The RAH originated as a single vessel, which later bifurcated, in 13 hemispheres. The doubled vessels separately branched from ACA in 10 hemispheres (Figures 2, 3(d), and 3(e)). The doubled RAHs arose from two portions of ACA in 1 hemisphere (from A1 and at the junction of ACA-ACoA).

**Figure 4:**
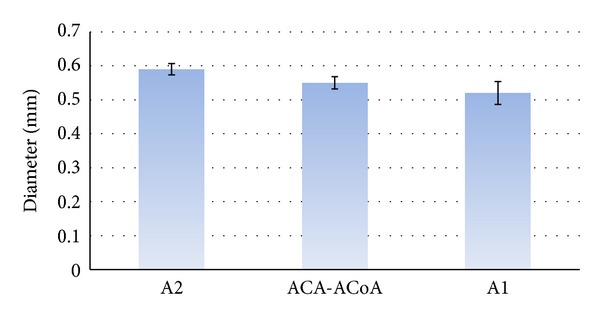
Comparison of the diameter (mean ± SEM) of the studied RAHs according to their origin from ACA.

The mean outer diameter of the RAH was 0.6 mm. The maximal measured diameter was 1.34 mm, and the minimal diameter was 0.19 mm. The mean diameter of the doubled vessels was 0.58 mm (from 0.97 to 0.23 mm). The mean outer diameter of the vessels originated from A2 was 0.59 mm with a maximal diameter 1.05 mm and minimal diameter 0.23 mm. The mean diameter of the vessels branched from A1 measured 0.52 mm with maximal and minimal values 0.68 and 0.3 mm, respectively. The mean diameter of the vessels, which originated at the junction of ACA-ACoA, was 0.55 mm with range 1.47 to 0.19 mm. The results are summarized in Tables 1 and 2 and Figure 4.

## 4. Discussion

The reports about the origin, the number, the course, the supplied territory, and the morphometric measures of the RAH are seldom in the literature.

The present report confirmed that the RAH is predominantly originating from A2 (47.81% of cases). The artery arose at the level of ACA-ACoA junction in 43.4% and from A1 in 3.55% of cases. The RAH was missing in 5.19% of cases. The anatomical study of Avci et al. on 62 hemispheres showed that the RAH branched from A2 in 64%, from the ACA-ACoA junction in 29%, and from A1 in 6% of cases. The artery was absent in 1.6% of cases [13]. The microsurgical report of Zunon-Kipré et al. concluded that A2 is the most common origin of RAH (58% of cases). The RAHs were more often originated from A1, in 30% of cases, than at the ACA-ACoA junction, in 12% of cases [3]. Similar conclusion was shown in the study of Perlmutter and Rhoton on 50 adult brains. The artery arose from A2 in 78%, from A1 in 14%, and at the level of ACoA in 8% of cases. The artery was missing in one hemisphere [14]. The RAH originated mainly from A2 (57% of cases) in the study of Gomes et al. on 30 unfixed brains. The artery arose at the level of ACA-ACoA junction in 35% and from A1 in 8% of cases. The RAH was absent in two hemispheres [15].

On the contrary, several authors reported the junction of ACA and ACoA to be the most frequent stem of the RAH. Loukas et al. presented a study containing 69 formalin-fixed hemispheres. The RAH was missing in 6% of cases. They reported that the artery originated mostly at the junction of ACA and ACoA in 62.3% of cases. In the remainder of cases with present artery, it originated from A2 in 23.3% and from A1 14.3% of cases [2]. The data from the microsurgical study of Tao et al. concluded that RAH branched mainly at the junction of ACA-ACoA with 46.88% of cases. The artery arose from A2 in 46.09% and from A1 in 7.03% of cases. The work was based on results from 90 hemispheres [9]. Uzün et al. found in their work based on 54 autopsy brains that the RAH originated at the junction of ACA-ACoA in 79.2%, from A2 in 14.6%, and from A1 in 6.2% of cases. The artery was missing in 6 hemispheres [16].

The anatomical studies of the anterior part of the circle of Willis are often reporting the presence of double RAH unilaterally or bilaterally. Gorczyca and Mohr reported double RAH in 48% of cases [17], Avci et al. in 22.6% of cases [13], and Tao et al. in 32.2% of cases. The study of Loukas et al. showed bilateral duplication of the RAH in 7% of cases [2]. Some researchers reported the presence of triple or even quadruple RAH in one hemisphere [6, 13, 15, 17]. In the present work, we did not observe more than two RAHs in one hemisphere. The artery was doubled in 6.28% of cases. It arose as one stem, which later bifurcated in 3.55%. Two arteries with a different origin were found in 2.7% of cases.

The value of the RAH diameter was highly variable in the literature, due to the use of different measuring procedures. In some cases, the microsurgical techniques with application of intravascular dyes were applied [9, 15]. In other cases calibrated digital pictures with software, which can calculate the number of pixels and convert them to metric measures, were used [2]. The difference in the values can be caused by using unfixed or formalin-fixed brains. The diameter was in the range from 0.2 to 2.9 mm; in a rare case it was as thick as A1 [10, 14]. The value of the mean outer diameter of all vessels in the current series was 0.6 mm with a range from 1.34 to 0.19 mm. The mean diameter of the double RAH was 0.58 mm with a range from 0.97 to 0.23 mm. Mostly one of the vessels was dominant with larger diameter.  Table 3 is presenting an overview of some morphological studies of the RAH in the last 10 years.

 Three possible courses of the RAH, before its penetrating the anterior perforating substance, are described in the literature. These courses were classified according to the relation of the RAH to the A1: type (I) or superior course, type (II) or anterior course, and type (III) or posterior course. Gomes et al. showed the superior course as the most common [15].

The RAH is a survivor of a series of anastomotic channels over and around the paleo-olfactorium between the ACA and the middle cerebral artery (MCA). The different organization of these channels can be the result of the variable origin, number, size, or course of the artery. The RAH is a branch from the primitive olfactory artery as the ophthalmic artery, the anterior choroid artery, and the MCA [3]. The artery is well developed at the twenty-fourth week of gestation [5].

The RAH usually originates few millimeters rostral or dorsal to the region of ACoA. This portion of the circle of Willis is the preferable area for aneurysm formation. The aneurysm of the ACoA presents about 30% of all cerebral aneurysms [7, 18]. Surgical procedures such as applying temporary clips in the anterior part of the circle of Willis or small excision of the gyrus rectus may cause damage or occlusion of the RAH. This may result in hemiparesis with brachial predominance and aphasia if the occluded artery is on the dominant side [2, 7]. The RAH lesions may also cause the paralysis of the face, palate and tongue, hemiplegia with brachial dominance, rarely severe weakness in upper limbs, and rigidity [1, 11]. Congenital factors may cause infarction of the RAH in infants [12]. The existence of multiple RAH was found to be associated with other cerebrovascular anomalies or malformations, which can cause complications in these patients [19]. According to some authors, the arteries with size ranged from 0.4 to 0.9 mm are subjected to the development of atheroma, which may be a possible cause of cerebral hemorrhage or infarctions [2, 3].

## 5. Conclusion

The RAH is commonly arising from A2 or at the ACA-ACoA junction. This portion of the circle of Willis is the place of many anatomical variations and malformations. The vessel can be absent, single, or multiple, and its diameter is highly variable. The awareness of these distinct anatomical and morphometric variations of the RAH is essential in planning the neurosurgical procedures in the anterior part of the circle of Willis to avoid the unexpected neurological complications.

## Figures and Tables

**Table 1:** The RAH presence in 366 hemispheres.

	Single vessel	Doubled vessels		Absent
		One origin	Two origins	
Hemispheres	324	13	10	19
%	88.5	3.55	2.7	5.19

**Table 2:** Origin of the RAH and its mean diameter ± the standard error of the mean (SEM).

	A1	ACA-ACoA junction	A2
Hemispheres	13 (3.55%)	159 (43.4%)	175 (47.81%)
Mean diameter (mm)	0.52 ± 0.034	0.55 ± 0.018	0.59 ± 0.016

**Table 3:** Summary of some anatomical studies of the RAH in the last 10 years.

	Number of hemispheres	Origin			Double RAH (%)	Mean diameter (mm)
		A2 (%)	ACA-ACoA (%)	A1 (%)		
Avci et al. (2003) [13]	62	64	29	8	22.6	0.45
Loukas et al. (2006) [2]	69	23.3	62.3	14.3	17	0.8
Tao et al. (2006) [9]	90	46.09	46.88	7.03	32.2	0.64
Uzün et al. (2009) [16]	108	14.6	79.2	6.2	—	0.67
Present study (2013)	366	47.81	43.4	3.55	6.28	0.6
